# Genome-Wide Analysis of Multidrug and Toxic Compound Extruction Transporters in Grape

**DOI:** 10.3389/fpls.2022.892638

**Published:** 2022-07-14

**Authors:** Megumi Watanabe, Shungo Otagaki, Shogo Matsumoto, Katsuhiro Shiratake

**Affiliations:** Laboratory of Horticultural Science, Graduate School of Bioagricultural Sciences, Nagoya University, Nagoya, Japan

**Keywords:** genome-wide analysis, grape, multidrug and toxic compound extraction transporter, MATE, secondary metabolite transporter, *Vitis vinifera*

## Abstract

Grape (*Vitis vinifera* L.) is an important fruit crop in the world. It is used as a table grape and is also used for raisin and wine production. Grape berries accumulate secondary metabolites, such as anthocyanins, tannins, and resveratrol, which are known as functional compounds for human health. Multidrug and toxic compound extrusion transporter (MATEs) transport secondary metabolites. MATEs also transport other solutes, including organic acids, and toxic xenobiotics, depending on cation gradient and play various roles in plants. MATE comprises 300–500 amino acid residues and possesses a MATE domain and 8–12 transmembrane domains. In the present study, 59 MATE genes were identified in the grape genome, and phylogenetic analysis revealed the presence of four groups of grape MATEs (Group 1–4). Their information, such as gene structures, protein motifs, predicted subcellular localizations, and gene IDs of four genome annotations, that is, CRIBI v1, CRIBI v2, Genoscope, and Vcost v3, were annotated. The transport substrates and physiological functions of grape MATEs were estimated based on their homology with the analyzed MATEs in other plant species. Group 1 may transport toxic compounds and alkaloids, Group 2 may transport polyphenolic compounds, Group 3 may transport organic acids, and Group 4 may transport plant hormones related to signal transduction. In addition to the known anthocyanin transporters, VvMATE37 and VvMATE39, a novel anthocyanin transporter, VvMATE38 in Group 2, was suggested as a key transporter for anthocyanin accumulation in grape berry skin. VvMATE46, VvMATE47, and VvMATE49 in Group 3 may contribute to Al^3+^ detoxification and Fe^2+^/Fe^3+^ translocation *via* organic acid transport. This study provides helpful and fundamental information for grape MATE studies and resolves the confusion of gene IDs in different genome annotations.

## Introduction

Grape (*Vitis vinifera* L.) is an important crop grown worldwide for table grape use and wine production. Grapes contain various secondary metabolites. For example, anthocyanins are responsible for the color of berries, catechins are responsible for the astringency of fruit taste, and resveratrol is responsible for disease resistance. These secondary metabolites also function as antioxidants and health-promoting compounds in humans. Plants produce these secondary metabolites and accumulate them strategically, defending against environmental stresses and pathogens.

The accumulation of secondary metabolites occurs *via* a series of synthetic enzymes and transport from the synthesis site to the accumulation site by the responsible transporters. Secondary metabolite transporters have been reported to belong to the following families (Nogia and Pati, 2021): ATP-binding cassette (ABC) transporter family (Rea, 2007), multidrug and toxic compound extrusion (MATE) transporter family (Omote et al., 2006), major facilitator superfamily (MFS; Nino-Gonzalez et al., 2019), NPF transporter family (Wen et al., 2020) and purine uptake permeases (PUPs; Dastmalchi et al., 2019). Some MATEs had been reported as antiporters using cation (H^+^/Na^+^) gradient and transport substrates from cytosol to apoplast or vacuole (Nogia and Pati, 2021). MATE comprises 300–500 amino acid residues and possesses a MATE domain and 8–12 transmembrane domains (Shoji, 2014). MATE is also called detoxification protein (DTX) in Arabidopsis (Li et al., 2002) and is involved in the detoxification mechanisms of toxic compounds and heavy metals (Diener et al., 2001; Li et al., 2002), tolerance to aluminum toxicity (Liu et al., 2009), iron homeostasis (Wang et al., 2017), and disease resistance (Ishihara et al., 2008).

MATEs are conserved in bacteria, fungi, animals, and plants (Hvorup et al., 2003). Genome-wide analyses, that is, identification and characterization of gene families using genome data, provide helpful and fundamental information on gene families, including evolutionary history and diversity of gene families and their members, characteristics of encoded proteins, and their physiological functions. Genome-wide analysis of MATEs and gene numbers in plants had been reported, including 56 in *Arabidopsis thaliana* (Li et al., 2002), 53 in *Oryza sativa* (Tiwari et al., 2014), 71 in *Populus trichocarpa* (Li et al., 2017), 117 in soybean (Liu et al., 2016), 48 in potato (Li et al., 2019), 67 in tomato (Dos Santos et al., 2017), and 73 in *Linum usitassimum* (Ali et al., 2021).

In grapes, only VvMATE1 and VvMATE2, which transport proanthocyanidins (Perez-Diaz et al., 2014), and VvAM1 and VvAM3, which transport cyanidin 3-glycosides (Gomez et al., 2009), have been reported. The numbers and functions of other grape MATEs are still unclear. Therefore, this study performed a genome-wide analysis of grape MATEs. As a result, 59 genes encoding MATEs were identified in the grape genome, obtaining useful information, including the gene IDs of different genome annotations, gene and protein structures, protein motifs, and gene expression profiles. In addition, the physiological functions of some MATEs in grapes were discussed with their gene expressions and transport substrates were estimated based on the homology with the analyzed MATEs in other plant species.

## Materials and Methods

### Identification of MATEs in Grape Genome

To obtain candidates of grape MATEs, BLASTP search (*e* ≤ 0.1) was performed against the CRIBI v2.1 genome annotation based on the *V. vinifera* 12X.0 genome assembly (Adam-Blondon, 2014) in Phytozome v13^1^ (Goodstein et al., 2012) using the amino acid sequences of Arabidopsis MATEs (Li et al., 2002) as queries. Against obtained non-redundant candidates of grape MATEs, the presence of conserved MATE domain was confirmed by Pfam^2^ (El-Gebali et al., 2019) and by the Conserved Domain Database of NCBI (CDD,^3^ Shennan Lu et al., 2020). Among the candidates, the proteins with predicted MATE domain by both Pfam and CDD were identified as grape MATEs. Molecular weight and isoelectric point were estimated by ProtParam^4^ (Gasteiger et al., 2005). The number of transmembrane domains was predicted by the Simple Modular Architecture Research Tool (SMART,^5^ Letunic et al., 2021).

MATEs in other grape genome annotations, that is, the CRIBI v1, the Genoscope, and the Vcost.v3 were identified as follows. BLASTP was performed against the CRIBI v1 genome annotation based on the *V. vinifera* 12X.0 genome assembly in EnsemblPlants.^6^ Whole protein sequences of the Genoscope genome annotation based on the *V. vinifera* 12X.0 genome assembly were downloaded from URGI^7^ or those of the Vcost.v3 genome annotation based on the *V. vinifera* 12X.2 genome assembly (Canaguier et al., 2017) were downloaded from URGI^8^ and in house BLASTP was performed.

### Multiple Sequence Alignment and Phylogenetic Analysis

Amino acid sequences of MATEs of grape and other plant species (Li et al., 2002; Takanashi et al., 2014; Biala-Leonhard et al., 2021) were aligned using the ClustalW program.^9^ The phylogenetic tree was created by MEGA X (Kumar et al., 2018) using the neighbor-joining model.

### Gene Structure and Motif Analyses

Gene structure (exon–intron structure) was schematized using coding sequences (CDS) and genomic sequence of the grape MATEs by the Gene Structure Display Server (GSDS,^10^ Hu et al., 2015). The Multiple Expectation Maximization for Motif Elicitation (MEME,^11^ Bailey et al., 2009) was used to identify the conserved motifs in the amino acid sequences of grape MATEs. The parameters were set as follows: site distribution on zero or one occurrence per sequences, the maximum number of motifs was 12, and motif width is between 6 and 50.

### *In silico* Gene Expression Analysis

The gene expression data in various organs, tissues, and developmental stages of grape by microarray analysis were downloaded from Grape eFP Browser (Fasoli et al., 2012).^12^ The gene expression data were converted to base 10 logarithm and then heatmap was created by using HemI 1.0 (Deng et al., 2014).^13^

## Results

### Identification of MATEs in the Grape Genome

To identify MATEs in the grape genome, BLAST searches were performed against the grape genome assembly 12X.0 (Adam-Blondon, 2014) and genome annotation CRIBI v2.1 (Vitulo et al., 2014) using Arabidopsis MATEs as queries. Consequently, 78 genes were hit in the grape genome. The presence of MATE domains in the candidates was confirmed using domain search tools Pfam and CDD. As a result, two of the 78 hits did not have a motif predicted by both Pfam and CDD and were therefore excluded from further analyses. Fifty-eight candidates predicted to possess the MATE domain by both Pfam and CDD were identified as grape MATEs and analyzed in further study. The 18 candidates predicted by either Pfam or CDD to possess the MATE domain are listed in Supplementary Table S2.

The whole grape genome was sequenced and annotated in 2007 (Jaillon et al., 2007; Velasco et al., 2007). Since then, the sequence information was improved from 8X to 12X in 2009 (Adam-Blondon, 2014), and annotation was revised to CRIBI v1 (Jérôme et al., 2012), Genoscope, CRIBI v2 (Vitulo et al., 2014) and Vcost v3 (Canaguier et al., 2017). Different annotations were assigned different gene IDs, and they were not unified. This has led to confusion in grape research. Therefore, the corresponding gene IDs in the different annotations were linked in this study.

A BLAST search of the 58 MATE genes identified in CRIBI v2 to the genes in the three annotations (CRIBI v1, Genoscope, and Vcost v3) was performed. Most genes showed 1:1 correspondence; however, some genes corresponded to 2. These genes are discussed below.

Two genes (VIT_217s0000g02990 and VIT_217s0000g03000) in CRIBI v2 corresponded to one gene (GSVIVT01008369001) in Genoscope (Table 1), although these genes had a 1:1 correspondence in CRIBI v1 and Vcost v3. These suggest that the annotation of Genoscope may be miss annotation.

**Table 1:** List of the identified MATEs in grape genome.

	CRIBI v2			CRIBI v1	Genoscope	VCost.v3	Protein			
Name	Gene ID	Chromosome location	Transcripts	Gene ID	Gene ID	Gene ID	Size (AAs)	MW (D)	Pi	TMDs
VvMATE1	VIT_201s0011g04430	chr1:4021807..4024501 reverse	VIT_201s0011g04430.1	VIT_01s0011g04430	GSVIVT01011803001	Vitvi01g00370	482	51,649	6.4	10
VvMATE2	VIT_201s0011g04450	chr1:4032047..4035573 reverse	VIT_201s0011g04450.1	VIT_01s0011g04450	GSVIVT01011801001	Vitvi01g01962	482	52,662	7.9	12
			VIT_201s0011g04450.2	VIT_01s0011g04450	GSVIVT01011801001	Vitvi01g01962	486	52,874	7.4	11
			VIT_201s0011g04450.3	VIT_01s0011g04450	GSVIVT01011801001	Vitvi01g01962	397	43,694	8.5	10
			VIT_201s0011g04450.4	VIT_01s0011g04450	GSVIVT01011801001	Vitvi01g01962	415	45,262	6.9	9
			VIT_201s0011g04450.5							
			VIT_201s0011g04450.6	VIT_01s0011g04450	GSVIVT01011801001	Vitvi01g01962	316	34,476	5.8	8
VvMATE3	VIT_201s0011g04460	chr1:4036752..4039385 forward	VIT_201s0011g04460.1	VIT_01s0011g04460	GSVIVT01011800001	Vitvi01g00371	540	59,659	8.7	9
			VIT_201s0011g04460.2	VIT_01s0011g04460	GSVIVT01011800001	Vitvi01g00371	471	51,907	8.2	9
			VIT_201s0011g04460.3	VIT_01s0011g04460	GSVIVT01011800001	Vitvi01g00371	337	37,035	8.4	6
			VIT_201s0011g04460.4							
			VIT_201s0011g04460.5	VIT_01s0011g04460	GSVIVT01011800001	Vitvi01g00371	280	30,310	8.1	6
VvMATE4	VIT_211s0052g01510	chr11:19255115..19265006 forward	VIT_211s0052g01510.1	VIT_11s0052g01510	GSVIVT01029142001	Vitvi11g01285	497	53,985	8.3	12
			VIT_211s0052g01510.2	VIT_11s0052g01510	GSVIVT01029142001	Vitvi11g01285	490	52,670	8.7	12
VvMATE5	VIT_211s0052g01540	chr11:19281101..19288934 forward	VIT_211s0052g01540.1	VIT_11s0052g01540	GSVIVT01029136001	Vitvi11g01286	457	49,183	8.7	11
			VIT_211s0052g01540.2							
			VIT_211s0052g01540.3	VIT_11s0052g01540	GSVIVT01029136001	Vitvi11g01286	412	44,652	7.7	9
			VIT_211s0052g01540.4	VIT_11s0052g01540	GSVIVT01029136001	Vitvi11g01286	339	36,786	8.2	8
			VIT_211s0052g01540.5	VIT_11s0052g01540	GSVIVT01029136001	Vitvi11g01286	273	29,571	8.9	6
			VIT_211s0052g01540.7							
			VIT_211s0052g01540.6	VIT_11s0052g01540	GSVIVT01029136001	Vitvi11g01286	310	33,718	8.4	7
VvMATE6	VIT_211s0052g01560	chr11:19292964..19306510reverse	VIT_211s0052g01560.1	VIT_11s0052g01570	GSVIVT01029131001	Vitvi11g01693	492	53,534	9.1	12
			VIT_211s0052g01560.3	VIT_11s0052g01570	GSVIVT01029131001	Vitvi11g01693	490	53,351	9.1	12
			VIT_211s0052g01560.4	VIT_11s0052g01570	GSVIVT01029131001	Vitvi11g01693	472	51,411	9.1	12
VvMATE7	VIT_211s0052g01560	chr11:19292964..19306510reverse	VIT_211s0052g01560.2	VIT_11s0052g01560	GSVIVT01029132001	Vitvi11g01692	490	52,734	8.6	12
			VIT_211s0052g01560.5	VIT_11s0052g01560	GSVIVT01029132001	Vitvi11g01692	471	50,692	8.6	10
			VIT_211s0052g01560.6	VIT_11s0052g01560	GSVIVT01029132001	Vitvi11g01692	497	54,015	8.1	12
			VIT_211s0052g01560.7	VIT_11s0052g01560	GSVIVT01029132001	Vitvi11g01692	438	47,079	8.8	9
			VIT_211s0052g01560.8	VIT_11s0052g01560	GSVIVT01029132001	Vitvi11g01692	478	51,972	8.1	10
VvMATE8	VIT_214s0006g02260	chr14:19602250..19605203 forward	VIT_214s0006g02260.1	VIT_14s0006g02260	GSVIVT01030951001	Vitvi14g01062	495	53,316	8.5	11
VvMATE9	VIT_217s0000g02970	chr17:2744209..2747536 reverse	VIT_217s0000g02970.1	VIT_17s0000g02970	GSVIVT01008370001	Vitvi17g00256	494	53,411	8.4	12
VvMATE10	VIT_217s0000g02990	chr17:2768313..2770594 reverse	VIT_217s0000g02990.1	VIT_17s0000g02990	GSVIVT01008369001	Vitvi17g00258	453	49,198	8.4	11
			VIT_217s0000g02990.2	VIT_17s0000g02990	GSVIVT01008369001	Vitvi17g00258	306	33,421	8.4	7
			VIT_217s0000g02990.3	VIT_17s0000g02990	GSVIVT01008369001	Vitvi17g00258	414	44,963	7.0	10
VvMATE11	VIT_217s0000g03000	chr17:2780295..2782669 reverse	VIT_217s0000g03000.1	VIT_17s0000g03000	GSVIVT01008369001	Vitvi17g00259	475	51,780	6.7	11
VvMATE12	VIT_217s0000g09270	chr17:10859683..10862639 forward	VIT_217s0000g09270.1	VIT_17s0000g09270	GSVIVT01007649001	Vitvi17g00915	449	48,804	8.6	10
VvMATE13	VIT_219s0014g02440	chr19:2532311..2534460 forward	VIT_219s0014g02440.1	VIT_19s0014g02440	GSVIVT01014308001	Vitvi19g00205	494	53,687	8.2	12
VvMATE14	VIT_219s0014g02450	chr19:2535101..2537063 forward	VIT_219s0014g02450.1	VIT_19s0014g02450	GSVIVT01014309001	Vitvi19g01864	488	53,438	7.1	10
VvMATE15	VIT_200s0225g00050	chrUn:14575577..14579088 reverse	VIT_200s0225g00050.1	VIT_00s0225g00050	GSVIVT01004076001	Vitvi01g00653	510	55,254	5.8	11
			VIT_200s0225g00050.2	VIT_00s0225g00050	GSVIVT01004076001	Vitvi01g00653	438	47,513	5.5	9
			VIT_200s0225g00050.3	VIT_00s0225g00050	GSVIVT01004076001	Vitvi01g00653	477	51,616	5.7	10
			VIT_200s0225g00050.4	VIT_00s0225g00050	GSVIVT01004076001	Vitvi01g00653	367	39,831	8.1	8
			VIT_200s0225g00050.5	VIT_00s0225g00050	GSVIVT01004076001	Vitvi01g00653	328	35,728	7.5	7
VvMATE16	VIT_200s0225g00060	chrUn:14591567..14594247 reverse	VIT_200s0225g00060.1	VIT_00s0225g00060	GSVIVT01004076001	Vitvi01g00655	513	55,893	6.6	9
			VIT_200s0225g00060.2							
			VIT_200s0225g00060.3	VIT_00s0225g00060	GSVIVT01004076001	Vitvi01g00655	472	51,231	6.3	8
			VIT_200s0225g00060.4	VIT_00s0225g00060	GSVIVT01004076001	Vitvi01g00655	455	49,811	7.6	8
VvMATE17	VIT_200s0225g00070	chrUn:14600470..14603490 reverse	VIT_200s0225g00070.1	VIT_00s0225g00070	GSVIVT01004077001	Vitvi01g00656	502	54,813	5.2	12
			VIT_200s0225g00070.2	VIT_00s0225g00070	GSVIVT01004077001	Vitvi01g00656	451	49,098	5.2	10
			VIT_200s0225g00070.3							
VvMATE18	VIT_200s0225g00080	chrUn:14613309..14616942 reverse	VIT_200s0225g00080.1	VIT_00s0225g00080	GSVIVT01004078001	Vitvi01g00657	517	56,521	8.8	10
VvMATE19	VIT_208s0056g00780	chr8:1221330..1226145 forward	VIT_208s0056g00780.1	VIT_08s0056g00780	GSVIVT01029901001	Vitvi08g00067	496	54,252	8.5	10
			VIT_208s0056g00780.2							
			VIT_208s0056g00780.3							
			VIT_208s0056g00780.4							
			VIT_208s0056g00780.5	VIT_08s0056g00780	GSVIVT01029901001	Vitvi08g00067	402	43,856	8.7	8
			VIT_208s0056g00780.6	VIT_08s0056g00780	GSVIVT01029901001	Vitvi08g00067	410	44,972	8.3	8
			VIT_208s0056g00780.7	VIT_08s0056g00780	GSVIVT01029901001	Vitvi08g00067	326	36,035	6.7	8
VvMATE20	VIT_208s0056g00870	chr8:1350357..1364245 reverse	VIT_208s0056g00870.1	VIT_08s0056g00870	GSVIVT01029912001	Vitvi08g00076	498	54,731	6.5	10
			VIT_208s0056g00870.2	VIT_08s0056g00870	GSVIVT01029912001	Vitvi08g00076	402	44,230	6.9	8
			VIT_208s0056g00870.3	VIT_08s0056g00870	GSVIVT01029912001	Vitvi08g00076	532	58,967	8.5	10
VvMATE21	VIT_208s0056g00890	chr8:1389528..1393841 reverse	VIT_208s0056g00890.1	VIT_08s0056g00890	GSVIVT01029913001	Vitvi08g00078	433	47,541	8.8	9
VvMATE22	VIT_208s0056g01000	chr8:1489979..1498340 forward	VIT_208s0056g01000.1	VIT_08s0056g01000	GSVIVT01029920001	Vitvi08g00085	485	53,483	7.5	10
VvMATE23	VIT_208s0056g01040	chr8:1508050..1531663 reverse	VIT_208s0056g01040.1	VIT_08s0056g01040	GSVIVT01029922001	Vitvi08g00086	486	53,540	9.3	12
			VIT_208s0056g01040.2	VIT_08s0056g01040	GSVIVT01029922001	Vitvi08g00086	409	45,181	9.2	10
			VIT_208s0056g01040.3	VIT_08s0056g01040	GSVIVT01029922001	Vitvi08g00086	455	50,007	9.3	11
VvMATE24	VIT_208s0056g01070	chr8:1548460..1560413 forward	VIT_208s0056g01070.1	VIT_08s0056g01070	GSVIVT01029926001	Vitvi08g00091	486	53,395	6.4	12
			VIT_208s0056g01070.2	VIT_08s0056g01070	GSVIVT01029926001	Vitvi08g00091	402	43,963	6.3	10
VvMATE25	VIT_208s0056g01080	chr8:1571971..1580783 forward	VIT_208s0056g01080.1	VIT_08s0056g01080	GSVIVT01029927001	Vitvi08g00092	514	56,571	8.2	12
VvMATE26	VIT_208s0056g01120	chr8:1700737..1717715 forward	VIT_208s0056g01120.1	VIT_08s0056g01120	GSVIVT01029934001	Vitvi08g00099	486	54,043	8.0	12
			VIT_208s0056g01120.2							
			VIT_208s0056g01120.3							
			VIT_208s0056g01120.4							
			VIT_208s0056g01120.5							
			VIT_208s0056g01120.6	VIT_08s0056g01120	GSVIVT01029934001	Vitvi08g00099	402	44,533	8.3	10
			VIT_208s0056g01120.7							
			VIT_208s0056g01120.8	VIT_08s0056g01120	GSVIVT01029934001	Vitvi08g00099	388	43,058	6.4	10
VvMATE27	VIT_210s0116g01860	chr10:1119912..1125200 forward	VIT_210s0116g01860.1	VIT_10s0116g01860	GSVIVT01012737001	Vitvi10g00107	505	54,449	5.1	10
			VIT_210s0116g01860.2	VIT_10s0116g01860	GSVIVT01012737001	Vitvi10g00107	493	53,154	5.4	10
VvMATE28	VIT_210s0116g01870	chr10:1127233..1137841 forward	VIT_210s0116g01870.1	VIT_10s0116g01870	GSVIVT01012739001	Vitvi10g00109	511	55,202	8.9	11
VvMATE29	VIT_210s0116g01880	chr10:1146808..1151516 forward	VIT_210s0116g01880.1	VIT_10s0116g01880	GSVIVT01012741001	Vitvi10g01644	510	54,854	6.9	12
			VIT_210s0116g01880.2	VIT_10s0116g01880	GSVIVT01012741001	Vitvi10g01644	399	42,734	7.5	10
			VIT_210s0116g01880.3	VIT_10s0116g01880	GSVIVT01012741001	Vitvi10g01644	373	40,489	7.6	8
			VIT_210s0116g01880.4	VIT_10s0116g01880	GSVIVT01012741001	Vitvi10g01644	425	45,331	7.6	9
			VIT_210s0116g01880.5	VIT_10s0116g01880	GSVIVT01012741001	Vitvi10g01644	321	34,764	6.8	6
VvMATE30	VIT_212s0028g01150	chr12:1714190..1716866 reverse	VIT_212s0028g01150.1	VIT_12s0028g01150	GSVIVT01020808001	Vitvi12g00099	505	54,898	8.4	11
			VIT_212s0028g01150.2	VIT_12s0028g01150	GSVIVT01020808001	Vitvi12g00099	486	52,782	8.6	11
			VIT_212s0028g01150.3	VIT_12s0028g01150	GSVIVT01020808001	Vitvi12g00099	378	41,544	8.9	8
VvMATE31	VIT_212s0028g01160	chr12:1739731..1742612 forward	VIT_212s0028g01160.1	VIT_12s0028g01160	GSVIVT01020806001	Vitvi12g00101	507	54,416	7.0	11
VvMATE32	VIT_212s0059g02160	chr12:6947541..6952813 forward	VIT_212s0059g02160.1	VIT_12s0059g02160	GSVIVT01030589001	Vitvi12g00506	553	59,506	8.0	12
			VIT_212s0059g02160.2	VIT_12s0059g02160	GSVIVT01030589001	Vitvi12g00506	517	55,496	8.3	11
			VIT_212s0059g02160.3	VIT_12s0059g02160	GSVIVT01030589001	Vitvi12g00506	470	50,236	8.6	10
VvMATE33	VIT_212s0059g02180	chr12:6968887..6971785 forward	VIT_212s0059g02180.1	VIT_12s0059g02180	GSVIVT01030590001	Vitvi12g02409	508	54,795	8.2	12
VvMATE34	VIT_212s0059g02220	chr12:7024881..7027760 forward	VIT_212s0059g02220.1	VIT_12s0059g02220	GSVIVT01030594001	Vitvi12g02411	508	54,630	8.0	12
VvMATE35	VIT_212s0059g02230	chr12:7030290..7041662 forward	VIT_212s0059g02230.1	VIT_12s0059g02230	GSVIVT01030595001	Vitvi12g00514	689	75,281	8.8	11
VvMATE36	VIT_215s0046g01800	chr15:18639468..18642895 reverse	VIT_215s0046g01800.1	VIT_15s0046g01800	GSVIVT01027003001	Vitvi15g01057	484	52,799	5.7	11
VvMATE37	VIT_216s0050g00900	chr16:17819177..17822079 reverse	VIT_216s0050g00900.1	VIT_16s0050g00900	GSVIVT01028885001	Vitvi16g01911	490	53,488	5.9	11
VvMATE38	VIT_216s0050g00910	chr16:17827694..17830625 reverse	VIT_216s0050g00910.1	VIT_16s0050g00910	GSVIVT01028882001	Vitvi16g01913	490	53,458	5.9	11
VvMATE39	VIT_216s0050g00930	chr16:17837264..17840061 reverse	VIT_216s0050g00930.1	VIT_16s0050g00930	GSVIVT01028879001	Vitvi16g01915	494	53,726	6.0	12
VvMATE40	VIT_218s0001g06790	chr18:5062242..5066242 reverse	VIT_218s0001g06790.1	VIT_18s0001g06790	GSVIVT01009104001	Vitvi18g00470	491	53,580	6.3	9
VvMATE41	VIT_218s0001g06820	chr18:5078186..5081480 reverse	VIT_218s0001g06820.1	VIT_18s0001g06820	GSVIVT01009105001	Vitvi18g00472	337	37,276	6.4	8
			VIT_218s0001g06820.2							
			VIT_218s0001g06820.3							
			VIT_218s0001g06820.4	VIT_18s0001g06820	GSVIVT01009105001	Vitvi18g00472	415	45,369	6.3	9
			VIT_218s0001g06820.5	VIT_18s0001g06820	GSVIVT01009105001	Vitvi18g00472	447	48,779	6.6	12
VvMATE42	VIT_218s0001g11760	chr18:10039981..10043834 reverse	VIT_218s0001g11760.1	VIT_18s0001g11760	GSVIVT01009629001	Vitvi18g00899	480	51,489	6.0	12
VvMATE43	VIT_203s0038g00410	chr3:388733..396945 reverse	VIT_203s0038g00410.1	VIT_03s0038g00410	GSVIVT01024199001	Vitvi03g00032	469	50,398	6.0	7
			VIT_203s0038g00410.2	VIT_03s0038g00410	GSVIVT01024199001	Vitvi03g00032	305	32,723	8.4	6
			VIT_203s0038g00410.3	VIT_03s0038g00410	GSVIVT01024199001	Vitvi03g00032	432	46,222	6.0	7
			VIT_203s0038g00410.4	VIT_03s0038g00410	GSVIVT01024199001	Vitvi03g00032	387	41,498	6.0	6
			VIT_203s0038g00410.5	VIT_03s0038g00410	GSVIVT01024199001	Vitvi03g00032	372	39,774	6.2	6
			VIT_203s0038g00410.6							
			VIT_203s0038g00410.7							
			VIT_203s0038g00410.8	VIT_03s0038g00410	GSVIVT01024199001	Vitvi03g00032	264	28,600	6.2	4
VvMATE44	VIT_203s0038g00430	chr3:398947..406451 reverse	VIT_203s0038g00430.1	VIT_03s0038g00430	GSVIVT01024198001	Vitvi03g00033	568	61,094	8.7	9
			VIT_203s0038g00430.2	VIT_03s0038g00430	GSVIVT01024198001	Vitvi03g00033	517	55,389	8.3	9
			VIT_203s0038g00430.3	VIT_03s0038g00430	GSVIVT01024198001	Vitvi03g00033	395	42,682	9.4	8
VvMATE45	VIT_203s0038g03200	chr3:2314225..2331028 reverse	VIT_203s0038g03200.1	VIT_03s0038g03200	GSVIVT01023938001	Vitvi03g00199	605	64,523	8.9	8
			VIT_203s0038g03200.2							
			VIT_203s0038g03200.3							
VvMATE46	VIT_206s0004g02140	chr6:2592369..2595923 forward	VIT_206s0004g02140.1	VIT_06s0004g02140	GSVIVT01025266001	Vitvi06g00216	514	54,418	6.7	8
VvMATE47	VIT_208s0007g08200	chr8:21562632..21567871 forward	VIT_208s0007g08200.1	VIT_08s0007g08200	GSVIVT01033317001	Vitvi08g01879	508	54,828	9.1	9
			VIT_208s0007g08200.3							
			VIT_208s0007g08200.2	VIT_08s0007g08200	GSVIVT01033317001	Vitvi08g01879	495	53,368	9.0	9
			VIT_208s0007g08200.5							
			VIT_208s0007g08200.4	VIT_08s0007g08200	GSVIVT01033317001	Vitvi08g01879	457	49,079	8.7	8
			VIT_208s0007g08200.6							
			VIT_208s0007g08200.7	VIT_08s0007g08200	GSVIVT01033317001	Vitvi08g01879	423	46,270	9.1	7
			VIT_208s0007g08200.8	VIT_08s0007g08200	GSVIVT01033317001	Vitvi08g01879	368	39,694	9.2	6
			VIT_208s0007g08200.9	VIT_08s0007g08200	GSVIVT01033317001	Vitvi08g01879	300	32,162	9.8	8
			VIT_208s0007g08200.10	VIT_08s0007g08200	GSVIVT01033317001	Vitvi08g01879	336	36,597	6.4	4
			VIT_208s0007g08200.11	VIT_08s0007g08200	GSVIVT01033317001	Vitvi08g01879	262	28,382	6.6	3
VvMATE48	VIT_208s0058g00510	chr8:9585099..9604413forward	VIT_208s0058g00510.1	VIT_08s0058g00510	GSVIVT01030279001	Vitvi08g00777	559	58,972	6.7	9
			VIT_208s0058g00510.2	VIT_08s0058g00510	GSVIVT01030279001	Vitvi08g00777	537	56,405	7.1	9
			VIT_208s0058g00510.3	VIT_08s0058g00510	GSVIVT01030279001	Vitvi08g00777	514	54,232	6.5	7
			VIT_208s0058g00510.4							
			VIT_208s0058g00510.5	VIT_08s0058g00510	GSVIVT01030279001	Vitvi08g00777	437	45,952	8.7	5
			VIT_208s0058g00510.6							
			VIT_208s0058g00510.7							
			VIT_208s0058g00510.8	VIT_08s0058g00510	GSVIVT01030279001	Vitvi08g00777	356	37,529	8.9	3
VvMATE49	VIT_213s0064g00930	chr13:22791692..22797039 forward	VIT_213s0064g00930.1	VIT_13s0064g00930	GSVIVT01032092001	Vitvi13g01734	543	58,180	9.0	10
			VIT_213s0064g00930.2	VIT_13s0064g00930	GSVIVT01032092001	Vitvi13g01734	513	54,633	8.9	11
			VIT_213s0064g00930.3	VIT_13s0064g00930	GSVIVT01032092001	Vitvi13g01734	389	41,572	10.2	9
VvMATE50	VIT_202s0025g04420	chr2:3894150..3895743 reverse	VIT_202s0025g04420.1	VIT_02s0025g04420	GSVIVT01019858001	Vitvi02g00403	531	57,765	6.1	11
VvMATE51	VIT_202s0025g05110	chr2:4578076..4579483 forward	VIT_202s0025g05110.1	VIT_02s0025g05110	-	Vitvi02g00466	469	51,169	8.7	10
VvMATE52	VIT_205s0077g02090	chr5:1620999..1622613 forward	VIT_205s0077g02090.1	VIT_05s0077g02090	GSVIVT01035122001	Vitvi05g00031	501	54,607	8.4	11
VvMATE53	VIT_207s0031g00750	chr7:16911063..16912725 forward	VIT_207s0031g00750.1	VIT_07s0031g00750	GSVIVT01022141001	Vitvi07g01759	554	60,180	8.3	12
VvMATE54	VIT_208s0058g01170	chr8:10606149..10609176 forward	VIT_208s0058g01170.1	VIT_08s0058g01170	GSVIVT01030196001	Vitvi08g00847	430	46,313	8.9	9
			VIT_208s0058g01170.2	VIT_08s0058g01170	GSVIVT01030196001	Vitvi08g00847	511	55,455	7.5	10
VvMATE55	VIT_211s0016g03050	chr11:2454949..2456542 reverse	VIT_211s0016g03050.1	VIT_11s0016g03050	GSVIVT01015294001	Vitvi11g00259	531	57,181	8.4	10
VvMATE56	VIT_213s0067g02980	chr13:1598628..1602479 reverse	VIT_213s0067g02980.1	VIT_13s0067g02980	GSVIVT01032676001	Vitvi13g00176	492	53,381	8.4	8
VvMATE57	VIT_216s0100g00460	chr16:15919961..15921566 forward	VIT_216s0100g00460.1	VIT_16s0100g00460	GSVIVT01010621001	Vitvi16g00951	535	57,895	8.1	11
VvMATE58	VIT_218s0001g08200	chr18:6688710..6690565 reverse	VIT_218s0001g08200.1	VIT_18s0001g08200	GSVIVT01009253001	Vitvi18g00583	554	60,232	7.1	12
VvMATE59	VIT_218s0086g00180	chr18:17293925..17296105 forward	VIT_218s0086g00180.1	VIT_18s0086g00180	GSVIVT01036812001	Vitvi18g01376	473	50,513	8.1	9
			VIT_218s0086g00180.3							
			VIT_218s0086g00180.2	VIT_18s0086g00180	GSVIVT01036812001	Vitvi18g01376	501	53,721	6.1	9

Gene ID, chromosome location, transcripts, protein size, MW, Molecular Weight; Pi, Isoelectric point and TMDs, Number of Transmembrane Domains are listed. Gene IDs was collected from the four different gene annotations, i.e. CRIBI v1, CRIBI v2, Genoscope and VCost.v3. Information about protein and domains were based on the CRIBI v2 annotation.

VIT_211s0052g01560 in CRIBI v2 corresponded to two genes (VIT_11s0052g01570 and VIT_11s0052g01560) in CRIBI v1, two genes (GSVIVT01029131001 and GSVIVT01029132001) in Genoscope, and two genes (Vitvi11g01693 and Vitvi11g01692) in Vcost v3. In the CRIBI v2 annotation, for VIT_211s0052g01560, eight splicing variants (VIT_211s0052g01560.1–8) were suggested, and VIT_211s0052g01560.1, 3, 4, and VIT_211s0052g01560.2, 5, 6, 7, and 8 were derived from different genome positions (Supplementary Figure S1). VIT_211s0052g01560.1, 3, and 4 correspond to VIT_11s0052g0157 in CRIBI v1, GSVIVT01029131001 in Genoscope, and Vitvi11g01693 in Vcost v3. In contrast, VIT_211s0052g01560.2, 5, 6, 7, and 8 corresponded to VIT_11s0052g0156 in CRIBI v1, GSVIVT01029132001 in Genoscope, and Vitvi11g01692 in Vcost v3, respectively (Supplementary Table S1). From these results, we concluded that the CRIBI v2 annotation VIT_211s0052g01560 contains two MATE genes, and 59 genes encoding MATEs are present in the grape genome.

For the 59 identified grape MATEs, information was collected based on the 12X.0 genome assembly and CRIBI v2.1 annotation. The number of amino acid residues, molecular weight, isoelectric point, and the number of transmembrane domains of MATE proteins and splicing variants are summarized in Table 1. The predicted subcellular localizations are listed in Supplementary Table S1. The number of amino acids was 262–689, molecular weights were 28,382–75,281, isoelectric points were 5–10, and the number of transmembrane domains was 3–12.

### Phylogenetic Analysis and Nomenclature

**Figure 1:**
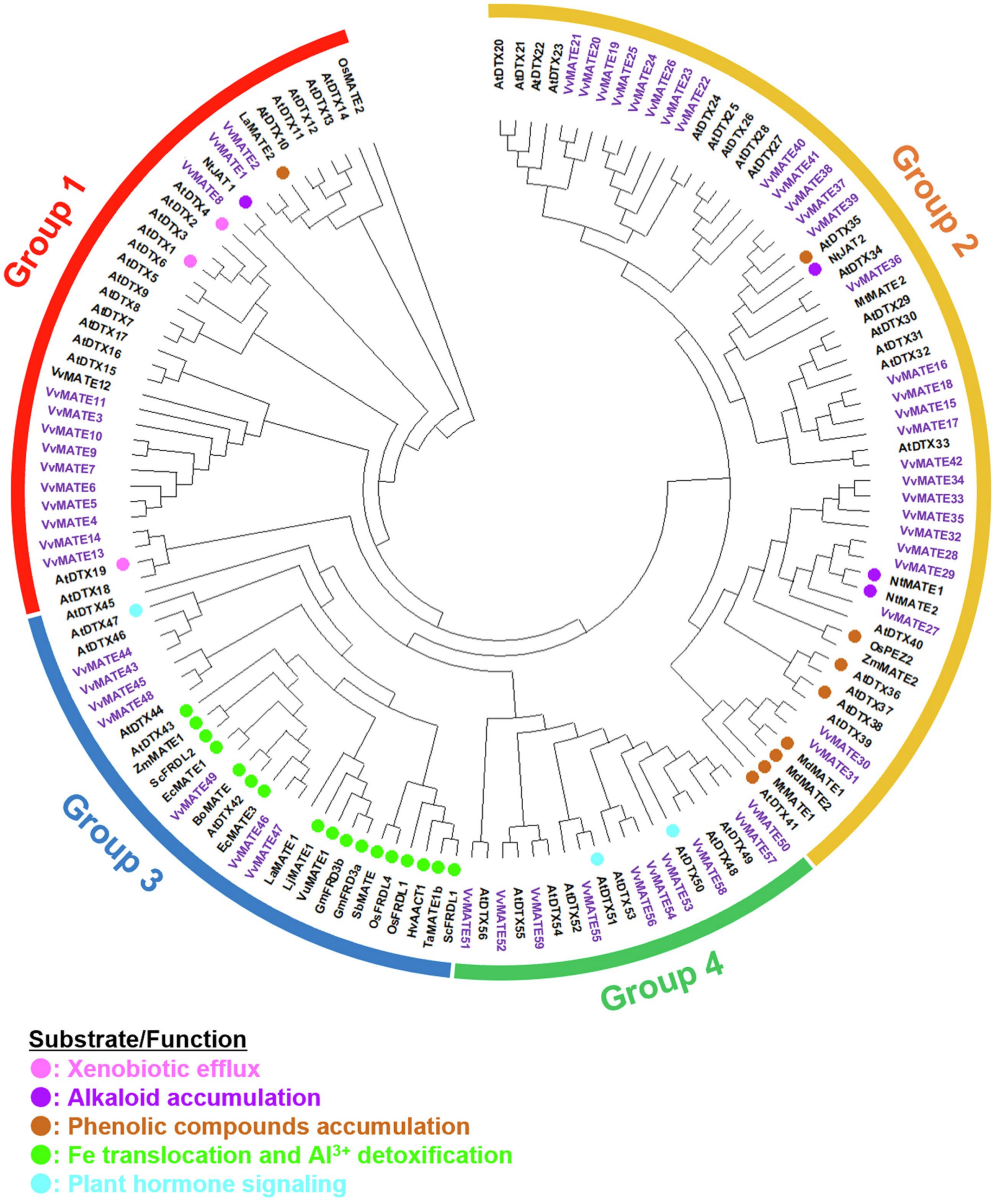
Phylogenetic tree of the MATEs of grape and those of other plant species reported previously. Amino acid sequences of the MATEs of grape and those of other plant species (Li et al., 2002; Takanashi et al., 2014; Biala-Leonhard et al., 2021) were aligned using the ClustalW program. The phylogenetic tree was created by MEGAX using the neighbor-joining model. The grape MATEs are indicated with purple and the MATEs of other plant species are indicated with circle (different colors show different physiological functions, Takanashi et al., 2014).

A phylogenetic tree of the amino acid sequences of 59 grape MATEs, 56 Arabidopsis MATEs, and the analyzed MATEs of various plants was generated (Figure 1). The tree revealed four groups of grape MATEs: 14 grape MATEs in Group 1, 28 in Group 2, 7 in Group 3, and 10 in Group 4. The 59 grape MATEs were named according to the gene ID in each group: VvMATE1-14 in Group 1, VvMATE15-42 in Group 2, VvMATE43-49 in Group 3, and VvMATE50-59 in Group 4 (Supplementary Table S1).

### Gene Structure and Protein Motif

**Figure 2:**
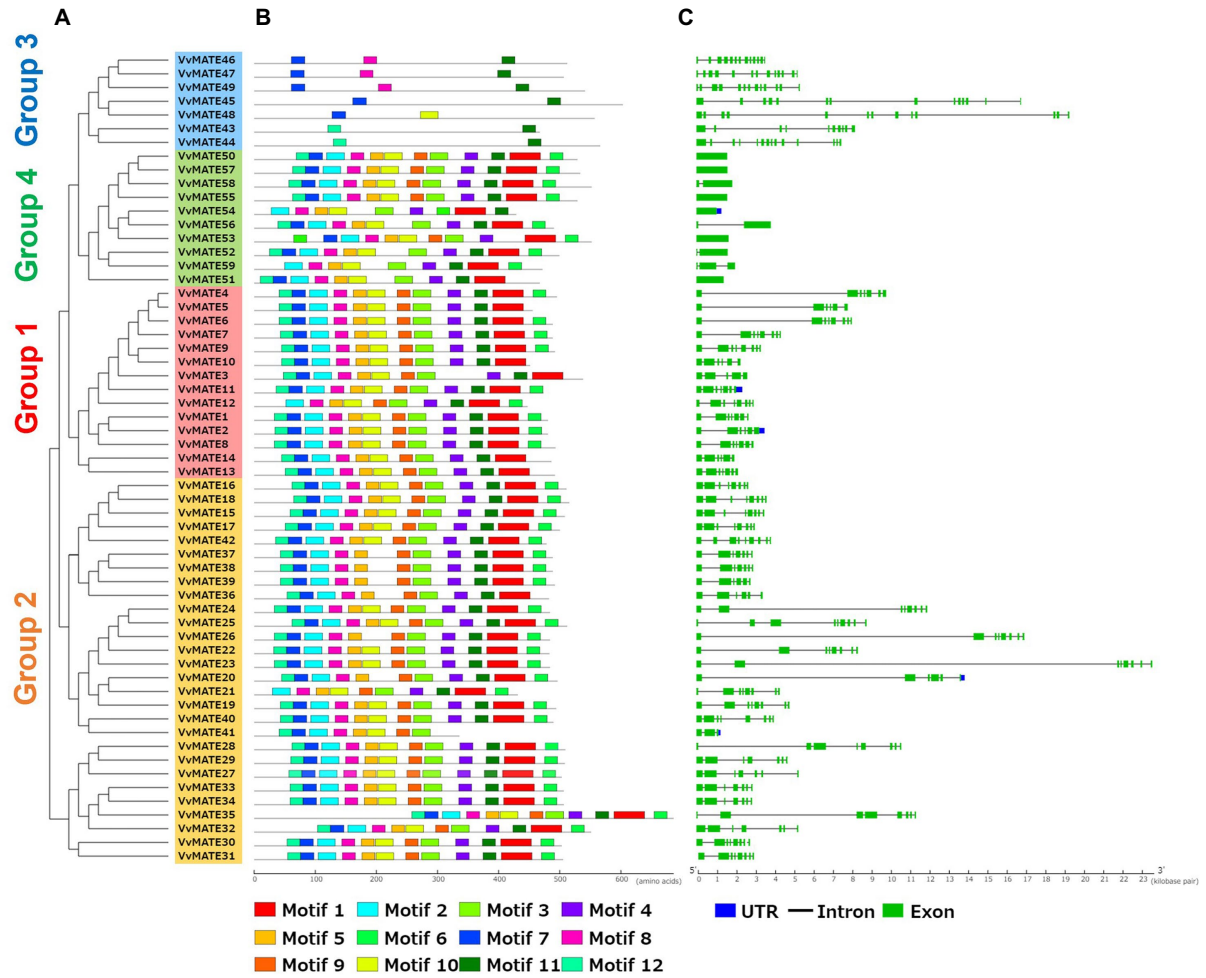
Conserved protein motifs and gene structures of the grape MATEs. **(A)** The neighbor-joining tree of Grape MATEs. **(B)** Protein motifs of the grape MATEs. Conserved motifs were identified by the MEME web server. Different motifs are represented by different colored boxes. **(C)** Exon–intron structures of the grape MATEs. Exons are shown as green boxes, introns are shown as black lines and untranslated regions (UTRs) are shown as blue boxes.

Exon-intron structures and protein motifs were shown with the phylogenetic tree generated by amino acid sequence homology (Figure 2A). The gene structures of grape MATEs were schematized in Figure 2C. The exon–intron structures were conserved in each group. The MATEs in group 1 had 5–8 exons and tended to have a long intron between the first and second exons. The MATEs in Group 2 have 7–9 exons, with long introns between the first and second exons and between the second and third exons. The MATEs in Group 3 had a larger number of exons than the other groups, that is, 10–13 exons. In contrast to Group 3, the MATEs in Group 4 have smaller exon numbers than the other groups, that is, 1–3 exons.

Protein motifs of grape MATEs were analyzed and schematized, as shown in Figure 2B. Twelve motifs were identified in grape MATEs. Generally, the motif patterns in Groups 1, 2, and 4 were similar. The motif patterns of Group 3 were different from those of the other groups, suggesting that the evolution and function of Group 3 were different from those of the other groups.

### Transport Substrate and Physiological Function

As for plant species, such as rice (Wang et al., 2016a) and flax (Ali et al., 2021), the grape MATEs were classified into four groups. Previous reports (Wang et al., 2016a) suggested that group members share characteristics and transport similar substrates.

Group 1 included AtDTX19, AtDTX1, and AtDTX6 in Arabidopsis, NtJAT1 in tobacco, and LaMATE2 in white lupin (Figure 1). AtDTX19 transports tetramethylammonium and protects the roots from toxic compounds in the soil (Diener et al., 2001). AtDTX1 transports alkaloids, antibiotics, and toxic compounds (Li et al., 2002). AtDTX6 transports paraquat and relates to paraquat tolerance (Xia et al., 2021). NtJAT1 transports alkaloids, such as nicotine (Morita et al., 2009). LaMATE2 is responsible for exporting isoflavonoids, such as genistein, from the roots to recruit Rhizobia (Biala-Leonhard et al., 2021). These results suggested that grape MATEs in Group 1 are involved in the transport of toxic compounds, alkaloids, and flavonoids. VvMATE1 and VvMATE2 have high homology with LaMATE2, suggesting that VvMATE1 and VvMATE2 have the potential to transport flavonoids.

Group 2 included AtDTX41, AtDTX35, AtDTX33, and AtDTX25 of Arabidopsis and NtJAT2, NtMATE1, and NtMATE2 of tobacco (Figure 1). AtDTX41 is responsible for accumulating proanthocyanidin precursors in vacuoles and for anthocyanin transport (Marinova et al., 2007). AtDTX35 is involved in flavonoid transport (Thompson et al., 2010), and also reported to be a chloride channel (Zhang et al., 2017). AtDTX33 also reported to be a chloride channel (Zhang et al., 2017). AtDTX25 transports ascorbate to vacuole and relates to Fe transport to seeds (Hoang et al., 2021). NtJAT2, NtMATE1, and NtMATE2 are involved in the transport of alkaloids, such as nicotine (Morita et al., 2009; Shoji et al., 2009; Shitan et al., 2014). Based on these reports, the grape MATEs in Group 2 are considered to transport flavonoids and alkaloids. The possibility that the grape MATEs in Group 2 were involved in the transport of polyphenolic compounds will be discussed later.

Group 3 included AtDTX43 and AtDTX43 in Arabidopsis and ZmMATE1 in maize (Figure 1). AtDTX43 contributes to iron and zinc homeostasis by transporting citric acid (Pineau et al., 2012). ZmMATE1 and AtDTX42 are responsible for the aluminum detoxification by transporting citrate (Maron et al., 2010; Wang et al., 2016b). These results suggested that the grape MATEs in Group 3 were responsible for organic acid transport.

Group 4 included AtDTX50 and AtDTX51 in Arabidopsis (Figure 1). AtDTX50 is responsible for the efflux of abscisic acid (ABA; Zhang et al., 2014). AtDTX51 has been reported to contribute to auxin and salicylic acid (SA) signaling (Sun et al., 2011; Li et al., 2014). These results suggested that some grape MATEs in Group 4 function in the transport or signaling of phytohormones, including ABA, auxin, and SA, and play an important role in the grapes’ growth and defense response.

### *In silico* Gene Expression Analysis

**Figure 3:**
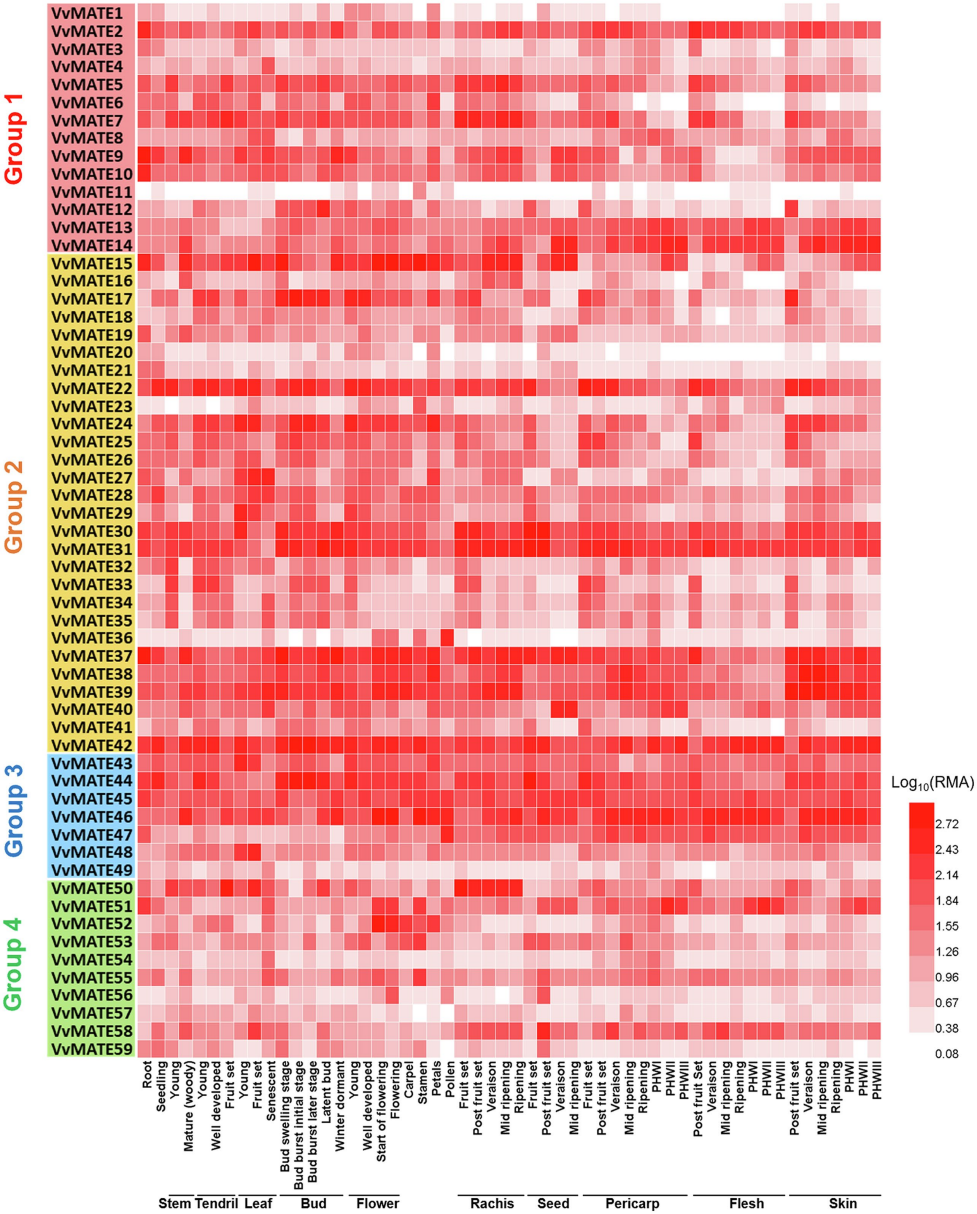
Gene expression profiles of the grape MATEs in various organs, tissues, and developmental stages. The gene expression data of *Vitis vinfera* “Corvina” (GEO Accession: GSE36128) were normalized using logarithm with the base of 10. The heat maps were created by HemI (Deng et al., 2014). PHWI: post-harvest withering I, PHWII: post-harvest withering II, PHWIII: berry flesh post-harvest withering III.

To understand the functions of MATEs in the growth and berry development of grapes, the gene expression in various organs, tissues, and growth stages of berries was profiled. Microarray data were obtained from the Grape eFP Browser, and a heat map was created (Figure 3). Although the genes of most grape MATEs were uniformly expressed in various organs, tissues, and growth stages of berries, some genes were expressed in specific organs, tissues, or growth stages of berries.

VvMATE31, VvMATE42, and VvMATE46 showed extremely high expression in all organs, tissues, and growth stages of berries, suggesting that they function as the major MATEs in grapes. VvMATE22 was highly expressed in seed and berry tissues at early developmental stages, and its expression decreased with development, suggesting that VvMATE22 function is important in young grape berries. VvMATE36 was highly expressed in pollen, and VvMATE50 expression was higher in the rachis than in other organs, although their expression was not specific.

## Discussion

Grapes are important fruits not only for eating but also for wine production. Polyphenolic compounds, including anthocyanins, catechins, and resveratrol, are important for the berries’ skin color and astringency and are functional compounds for human health. Therefore, grape berry content has a significant effect on grape quality.

**Figure 4:**
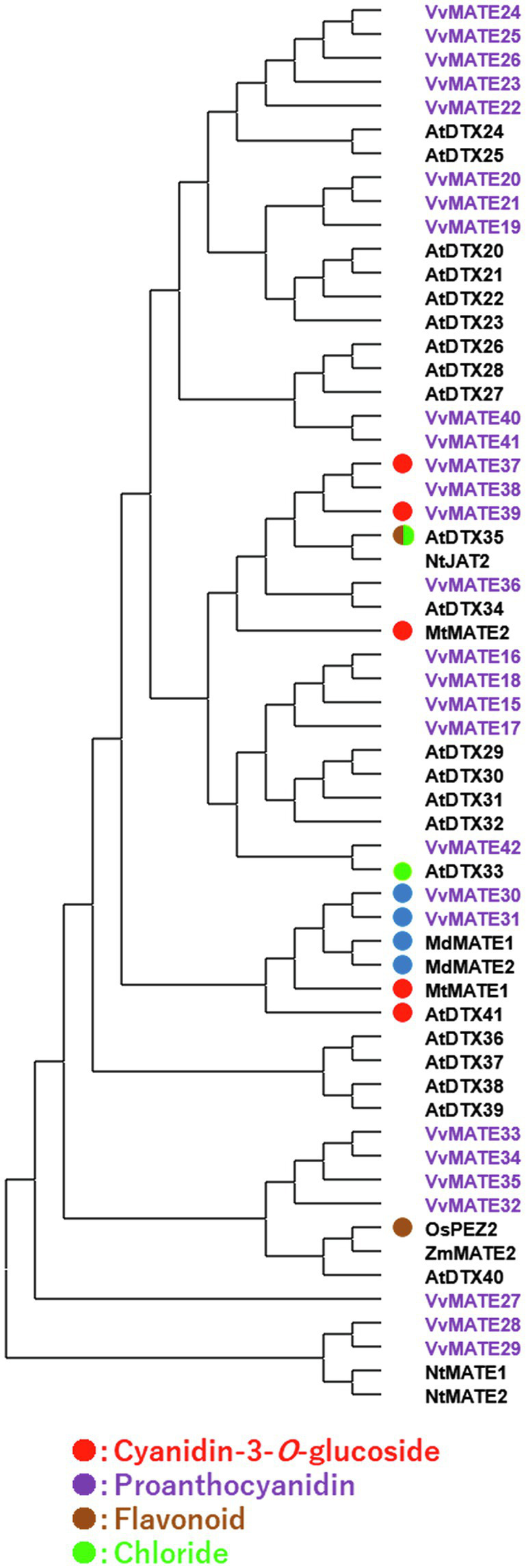
Phylogenetic tree of the group 2 MATEs of grape, Arabidopsis, and those of other plant species reported previously. Amino acid sequences of the MATEs of grape and those of other plant species (Li et al., 2002; Takanashi et al., 2014) were aligned using the ClustalW program. The phylogenetic tree was created by MEGAX using the neighbor-joining model. The group2 of grape MATEs are indicated with purple. Substrates of the MATEs with circles have been reported previously (different colors show different substrate, Takanashi et al., 2014; Zhang et al., 2017).

As mentioned above, grape MATEs belonging to Group 2 may potentially transport polyphenolic compounds. Indeed, VvMATE1 and VvMATE2 (VvMATE31 and VvMATE30 in this study) have been reported to transport proanthocyanidins (Perez-Diaz et al., 2014), and VvAM1 and VvAM3 (VvMATE39 and VvMATE37 in this study) have been reported to transport anthocyanins, that is, cyanidin 3-glycosides (Gomez et al., 2009). Uncharacterized VvMATE38 in the same clade as VvMATE37 and VvMATE39 (Figure 4) had the potential to transport anthocyanins. The gene expression of VvMATE38 increased dramatically at the veraison stage, whereas those of VvMATE37 and VvMATE39 did not (Supplementary Figure S2A). These results suggested that VvMATE38 functions as an anthocyanin transporter for grape berry skin coloration.

VvMATE3 and VvMATE1 (VvMATE30 and VvMATE 31 in this study) have been reported to transport proanthocyanidins. The gene expression levels of VvMATE3 and VvMATE1 in seeds, where proanthocyanidin accumulation occurs, were high before veraison and decreased rapidly at the veraison stage, whereas those in berry skin did not change before and after veraison (Perez-Diaz et al., 2014). These trends were consistent with the gene expression of VvMATE30 and VvMATE 31 in the *in silico* gene expression analysis shown in Supplementary Figure S2B. Based on these results, VvMATE30 and VvMATE31 were considered to play important roles in the accumulation of proanthocyanidins in grape seeds.

Aluminum ions (Al^3+^) in acidic soils, which account for approximately 50% of the world’s cultivated land, are toxic to most plants. In contrast, iron ions are essential for plants, and their homeostasis is important for various enzymatic reactions, including respiration and electron transfer systems in photosynthesis (Briat et al., 2015). Al^3+^ and iron ions are chelated by organic acids, such as citric acid and malic acid, produced by plants. Chelation is important for Al^3+^detoxification and Fe ion facilitation (Kochian et al., 2015). As mentioned above, the grape MATEs in Group 3 have a potential to detoxify Al^3+^ and promote the translocation of iron ions *via* organic acid transport. Protein motif analysis showed that the grape MATEs in Group 3 had a unique structural feature (Figure 2B). This structural feature has also been observed in soybeans (Liu et al., 2016), poplars (Li et al., 2017) and flax (Ali et al., 2021). This suggested that the structural features of the MATEs in Group 3 are important for the transport of organic acids.

**Figure 5:**
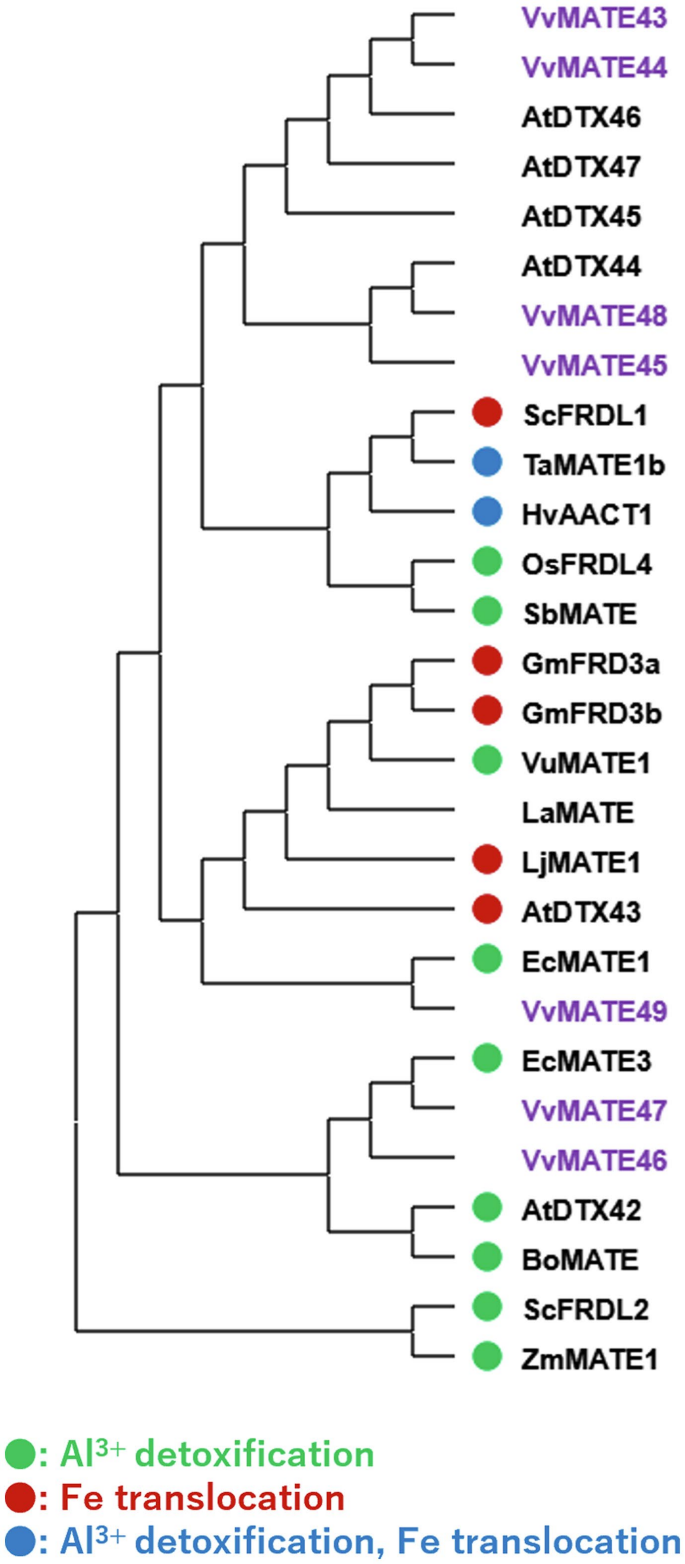
Phylogenetic tree of the group 3 MATEs of grape, Arabidopsis, and those of other plant species reported previously. Amino acid sequences of the MATEs of grape and those of other plant species (Li et al., 2002; Takanashi et al., 2014) were aligned using the ClustalW program. The phylogenetic tree was created by MEGAX using the neighbor-joining model. The group3 of grape MATEs are indicated with purple. MATEs with circle are reported their substrate previously (different colors show different substrates, Takanashi et al., 2014).

In the same clade, VvMATE 46, VvMATE 47, and VvMATE 49 were present with the MATEs of other plants, which are related to Al^3+^ detoxification or iron ion translocation *via* citrate transport (Figure 5). VvMATE46, VvMATE47, and VvMATE49 were expressed in the whole plant body, including roots (Figure 3). These results suggested that VvMATE46, VvMATE47, and VvMATE49 are involved in Al^3+^ detoxification in roots and iron ion translocation in the plant body. The gene expression of VvMATE46, VvMATE47, and VvMATE49 were detected in berries (Figure 3), suggesting that they have a potential to transport organic acids and play a role in organic acid accumulation in grape berries.

## Conclusion

This study performed a genome-wide analysis of grape MATEs and identified 59 genes in the grape MATE family. We collected and listed useful information, such as gene IDs, gene structures, protein sizes, motifs, predicted subcellular localization, and gene expression profiles. Phylogenetic analysis revealed the presence of four groups in the grape MATE family, and the transport substrates were estimated for each group. Physiological functions of grape MATEs were discussed together with the estimated transport substrates and gene expression profiles. From this discussion, the uncharacterized MATE, VvMATE38, was suggested as an anthocyanin transporter to color grape berry skin. Different genome annotations assigned different gene IDs for the grape genome, which led to confusion in grape research. In this study, the gene IDs by the different genome annotations were corresponded and the confusion was eliminated. The information provided in this study is essential for research on grape MATEs and will be useful in future studies.

## Data Availability Statement

The original contributions presented in the study are included in the article/Supplementary Material, further inquiries can be directed to the corresponding author.

## Author Contributions

KS conceived and managed the research project. MW performed analyses. SO and SM provided scientific suggestions. MW and KS wrote the manuscript. All authors read and approved the final manuscript.

## Funding

This work was supported by grants from the Japan Society for the Promotion of Science Grants-in-Aid for Scientific Research (24380019, 18H05361, 18H03950, 21H02184, and 21K19111) and by the research program on development of innovative technology grants (28001A and 28001AB) from the Project of the Bio-oriented Technology Research Advancement Institution (BRAIN).

## Conflict of Interest

The authors declare that the research was conducted in the absence of any commercial or financial relationships that could be construed as a potential conflict of interest.

## Publisher’s Note

All claims expressed in this article are solely those of the authors and do not necessarily represent those of their affiliated organizations, or those of the publisher, the editors and the reviewers. Any product that may be evaluated in this article, or claim that may be made by its manufacturer, is not guaranteed or endorsed by the publisher.

## Footnotes

^1^ https://phytozome-next.jgi.doe.gov/

^2^ http://pfam.xfam.org/

^3^ http://www.ncbi.nlm.nih.gov/Structure/cdd/wrpsb.cgi/

^4^ http://www.expasy.org/tools/protparam.html

^5^ http://smart.embl-heidelberg.de/smart/batch.pl

^6^ https://plants.ensembl.org/Vitis_vinifera/Tools/Blast

^7^ https://urgi.versailles.inra.fr/Species/Vitis/Annotations

^8^ https://urgi.versailles.inra.fr/Species/Vitis/Annotations

^9^ https://www.genome.jp/tools-bin/clustalw

^10^ http://gsds.gao-lab.org/

^11^ https://meme-suite.org/meme/tools/meme

^12^ http://bar.utoronto.ca/efp_grape/cgi-bin/efpWeb.cgi

^13^ http://hemi.biocuckoo.org/
